# Topoisomerase II Poisons for Glioblastoma; Existing Challenges and Opportunities to Personalize Therapy

**DOI:** 10.3389/fneur.2018.00459

**Published:** 2018-06-20

**Authors:** Amol Mehta, Chidiebere U. Awah, Adam M. Sonabend

**Affiliations:** Department of Neurological Surgery, Northwestern University Feinberg School of Medicine, Chicago, IL, United States

**Keywords:** topoisomerase 2 Poisons, glioblastoma multiforme, personalized therapy, drug delivery, tumor susceptibility

## Abstract

Despite advances in surgery, radiotherapy, and chemotherapy, glioblastoma (GBM) remains a malignancy with poor prognosis. The molecular profile of GBM is diverse across patients, and individual responses to therapy are highly variable. Yet, patients diagnosed with GBM are treated with a rather uniform paradigm. Exploiting these molecular differences and inter-individual responses to therapy may present an opportunity to improve the otherwise bleak prognosis of patients with GBM. This review aims to examine one group of chemotherapeutics: Topoisomerase 2 (TOP2) poisons, a class of drugs that enables TOP2 to induce DNA damage, but interferes with its ability to repair it. These potent chemotherapeutic agents are currently used for a number of malignancies and have shown promise in the treatment of GBM. Despite their robust efficacy *in vitro*, some of these agents have fallen short of achieving similar results in clinical trials for this tumor. In this review, we explore reasons for this discrepancy, focusing on drug delivery and individual susceptibility differences as challenges for effective TOP2-targeting for GBM. We critically review the evidence implicating genes in susceptibility to TOP2 poisons and categorize this evidence as experimental, correlative or both. This is important as mere experimental evidence does not necessarily lead to identification of genes that serve as good biomarkers of susceptibility for personalizing the use of these drugs.

## Introduction

Glioblastoma (GBM) is the most common primary type of brain malignancy, representing 28% of all Central Nervous System (CNS) tumors and 80% of the malignant subset (1). These tumors carry a dismal prognosis; the average survival for GBM patients is 13 months, with a 2-year survival of 27%, and a 5-year survival of 5.1% (2–4). All patients diagnosed with GBM undergo the same non-curative treatment, consisting of surgery, chemotherapy and radiation. This homogeneous treatment paradigm stands in stark contrast to the heterogeneous molecular profile found in GBM which has led to the classification of these tumors into subtypes based on their patterns of gene expression, genetic alterations, and DNA methylation (5, 6). These expression patterns and their underlying mechanisms could provide a unique tumoral vulnerability. These tumors are also rather unpredictable with regards to their response to therapies, and a growing body of literature is focused on the prediction of inter-individual response to treatment with the goal of personalizing therapies for GBM. This review aims to critically examine one such class of chemotherapeutics: Topoisomerase-2 (TOP2) poisons, one of the most powerful and common groups of chemotherapeutic agents used for cancer. We discuss these drugs given recent evidence suggesting they are highly effective for a subset of GBM, eliciting the idea that a refined assessment of their efficacy in select patient populations may show their value for treating this disease and provide a strategy for their use with a personalized or precision-medicine approach. In this context, we provide an overview of the literature that evaluates cancer susceptibility to TOP2-targeting drugs.

## TOP2 targeting drugs background

### Topoisomerase biology

**Figure 1:**
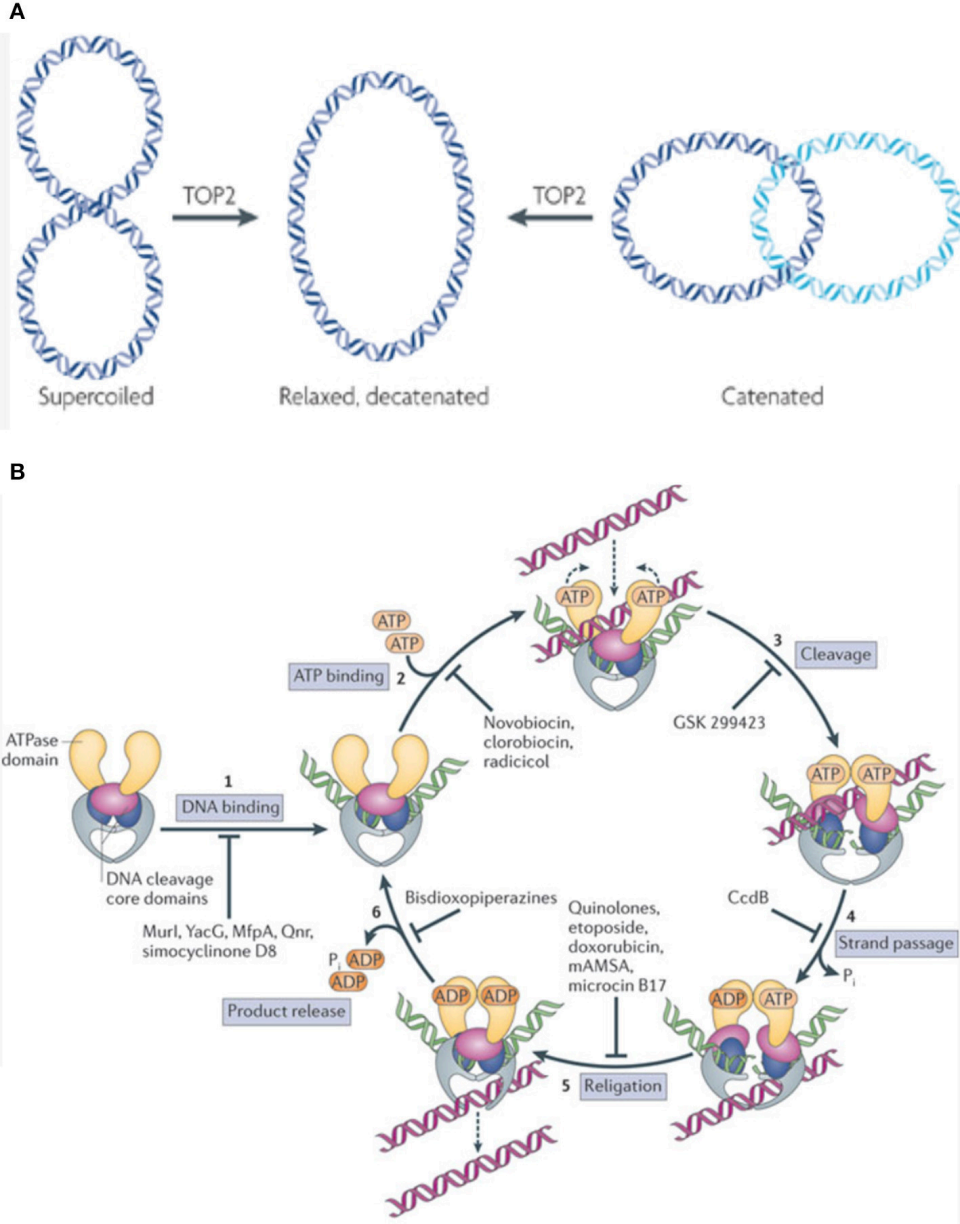
**(A)** TOP2 functions by relieving supercoils generated as a function of the double stranded nature of DNA. Additionally, TOP2 removes catenanes (interlinked DNA products) formed during replication. This allows the cell to carry out vital functions like replication, repair, and transcription. *Figure modified and reproduced with permission from Nature Publishing Group*. **(B)** TOP2 inhibitors function by interfering with various steps in the enzyme's catalytic cycle. Some Topoisomerase inhibitors work by inhibiting DNA binding, thus preventing the formation of the DNA-enzyme complex (1), while others act at the next step, preventing ATP from binding to the DNA-enzyme complex which in turn does not allow for the formation of a closed clamp structure (2). Some agents prevent the enzymatic generation of a double stranded DNA break (DSB) (3). Other downstream agents function by preventing the passage of the intact strand through the already generated ds-DNA break (4). Etoposide, doxorubicin, and analogous agents function by inhibiting DNA religation, thus stabilizing the DSB-enzyme complex and triggering apoptosis (5). Some inhibitors work at the last step of the catalytic cycle, and preventing the release of product from the enzyme (6). *Figure reproduced with permission from Nature Publishing Group*.

Topoisomerases are ubiquitously expressed enzymes that execute many crucial cellular functions. They are necessary, as the double helical nature of DNA leads to torsional forces that need to be relaxed for processes like DNA replication, repair, transcription, and chromosomal segregation (7). Also, by creating single or double stranded DNA breaks (DSB), topoisomerases remove supercoils generated by the continuous unwinding and rewinding of double stranded DNA (7, 8). These enzymes also perform decatenating functions, which are necessary to remove interlinked DNA products (catenanes) formed during replication (Figure 1) (7, 8).

There are two main categories of Topoisomerases, TOP1 and TOP2, each with their own subtypes (A/B), distinct functions, and mechanisms of action. TOP2 decoils DNA by creating transient DSB and passing a separate DNA duplex through the breaks generated, enhancing chromatin accessibility (9, 10). TOP2A/B differ in their catalytic sites; the etoposide binding site of hTOP2A contains a methionine (M762) residue while hTOP2B contains a Glutamine (Q778). This difference makes the DNA-TOP2A complex more stable than the DNA-TOP2B complex, and this stability has been shown to increase cellular susceptibility to TOP2 poisions (11, 12). TOP2A is typically expressed by cycling cells, whereas TOP2B is often expressed by post-mitotic cells (7). Both enzymes are capable of re-ligating the break they generate. The period between the cleaved and ligated states is when these enzymes are susceptible to TOP2 poisons (13).

### TOP2 targeting drugs' mechanism of action

TOP2 targeting drugs can be split into two broad categories; catalytic inhibitors and TOP2 poisons. Catalytic inhibitors impede an important catalytic step in the enzyme's reaction cycle (9, 8). Examples include Aclarubicin, which functions by preventing TOP2 from initially binding to DNA, and Merbarone, which prevents TOP2 from cleaving the DNA once bound (9).

Other drugs are called TOP2 poisons, as they kill cells by stabilizing transient intermediates in which the enzyme is linked to DNA and thus trigger DSB, essentially converting the topoisomerase enzyme into an agent that damages DNA, leading to apoptosis (14). Epipodophyllotoxins like etoposide perform the above functions by covalently linking to DNA while Anthracyclines do so by intercalating into DNA. Doxorubicin, an Anthracycline, is a TOP2 poison that intercalates into DNA and also dislodges histones from their chromatin, disrupting the normal DNA damage response, interfering with epigenetic regulation at damaged sites, and generating reactive oxygen species (15, 16).

Some agents, like dexrazoxane and other bisdiozopiperazines, inhibit TOP2 after it passes the duplex through its DNA break, but before it hydrolyzes ATP. This effectively prevents the closed-clamp structure of TOP2 from re-opening, and the enzyme from turning over (17).

## TOP2 poisons in brain tumors

### Pre-clinical and early clinical data

There is some evidence to suggest that the use of TOP2-targeting drugs may be efficacious in the treatment of GBM. Numerous *in vitro* and animal studies have demonstrated the anti-tumor effects of doxorubicin against GBM cell lines (18–21). A human *ex vivo* study investigating the response to TOP2 poisons in short-term cultures derived from malignant gliomas demonstrated that both etoposide and doxorubicin are toxic to these tumor cells (18), while another study in rat models designed to study combination TOP1 + TOP2 therapies showed doxorubicin toxicity toward GBM cell lines (19).

Phase II studies tested the use of systemic etoposide in recurrent gliomas and showed that a subset of recurrent GBM patients partially responded to an etoposide-containing regimen (22, 23). Other studies, however, demonstrated a lack of efficacy, which may be partially explained by variable expression of TOP2A within GBM. It is important to keep in mind some of these trials used metronomic doses of etoposide (35 mg/m^2^). This dose is sub-optimal given that the majority of trials that have demonstrated etoposide's efficacy against GBM have used doses of 50 mg/m^2^ and 100 mg/m^2^ (24). Additionally, many of these trials used etoposide in combination with a number of other agents (25). Additionally, a meta-analysis found that treatment with etoposide is associated with overall increased survival (24).

**Figure 2:**
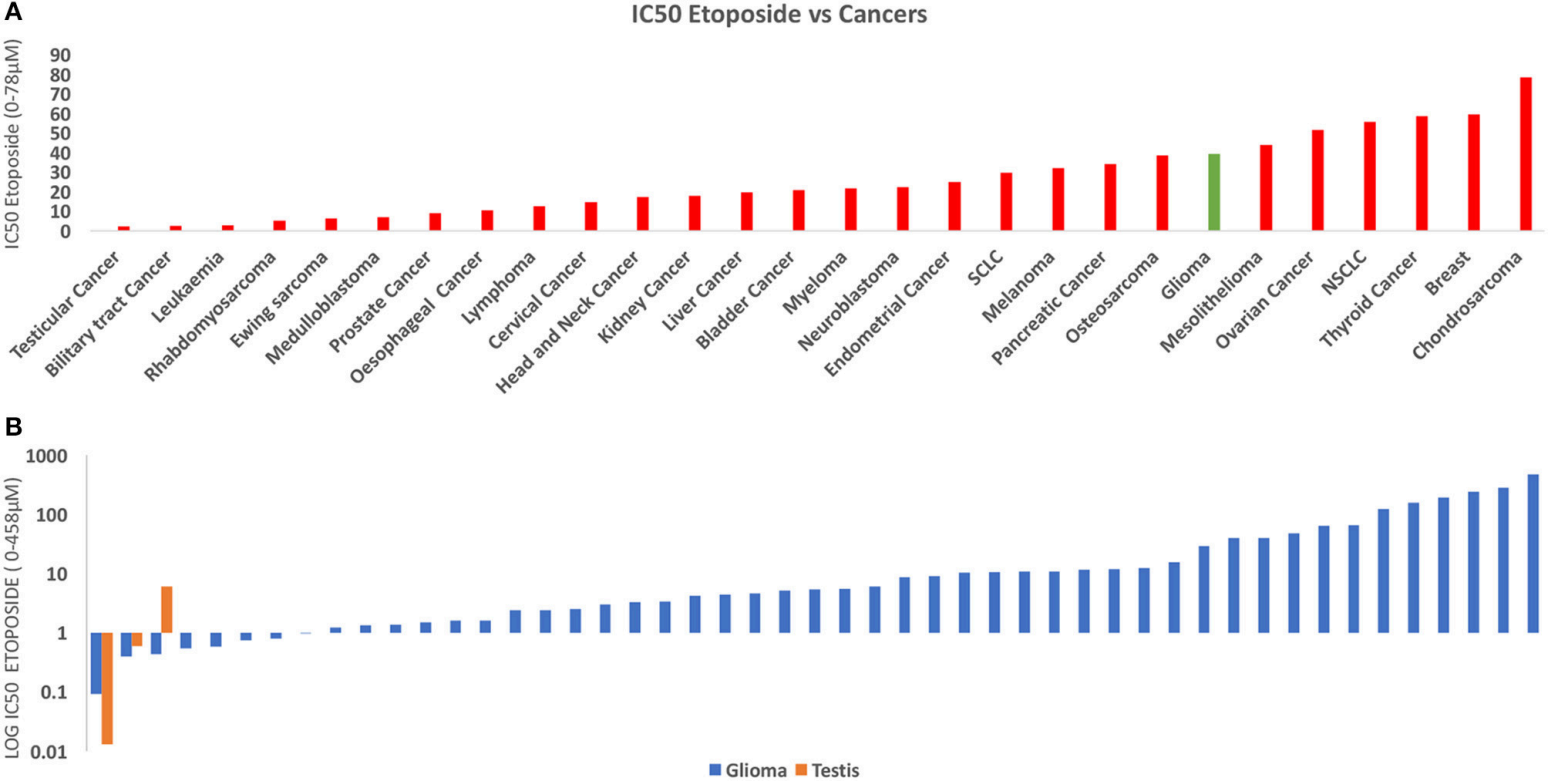
**(A)** This figure depicts the IC_50_ of Etoposide against human cancers derived from 900 cell lines. The data was derived from Cancerxgene. The IC_50_ for each cancer group was averaged and the standard deviation was then determined. Testicular cancer demonstrated the highest susceptibility to etoposide. The response of Glioma (red) was similar to many of these cell lines including SCLC and Osteosarcoma, both of which are traditionally treated with Etoposide (26). **(B)** Chart derived from the same data comparing IC50 Etoposide for Glioma (Orange) and Testicular Cancer (Blue). Some glioma cell lines demonstrate a similar response to etoposide as do testicular cancer cell lines.

To investigate the relative susceptibility of gliomas to etoposide compared to other cancers, we conducted an analysis and compared the susceptibility of 667 human cancer cell lines to etoposide using publicly available data from https://www.cancerrxgene.org (Figure 2) (27). Our analysis demonstrates that testicular cancer is the most responsive to etoposide, and gliomas' response is comparable to that of lymphoma, osteosarcoma, and neuroblastoma. We found gliomas had a similar response to etoposide as small cell lung cancer (SCLC) and myeloma, two cancers that have traditionally been treated with etoposide.

### Current challenges in TOP2-targeting therapy for brain tumors

While early data for TOP2 poisons is promising, their pharmacokinetic profile and poor blood-brain barrier (BBB) penetrance have limited their efficacy in the treatment of GBM. The underperformance of etoposide can be attributed to low levels and wide ranges of intra-tumoral drug concentrations. Concentrations have been shown to range between 12 and 36% of blood concentration, with intratumoral concentrations ranging between 2–6 μM (28–30). Conventional systemic delivery beyond this dosing is limited by toxicity. Similarly, the primary explanation for doxorubicin's disappointing efficacy *in vivo* has been its lack of ability to penetrate the BBB, due to its high molecular weight and low lipophilicity (31).

**Figure 3:**
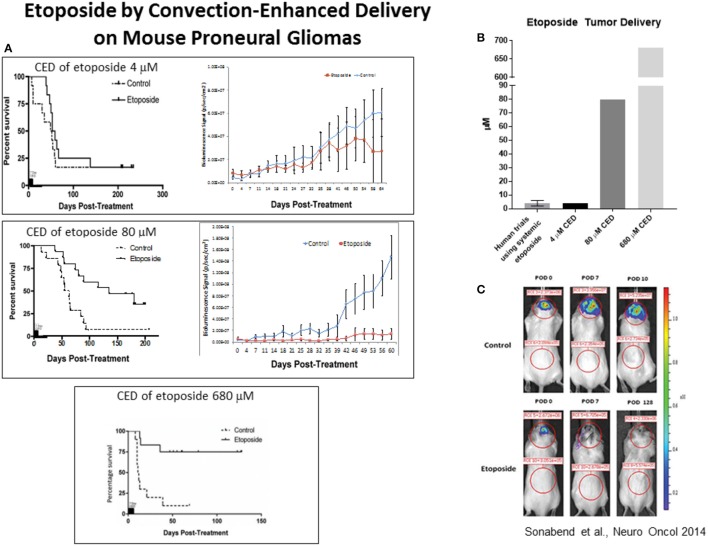
**(A)** In a recent study, we found that mice treated with 80 μM etoposide via Convection Enhanced Delivery (CED) demonstrated prolonged survival when compared control mice treated with 4 μM (the mean concentration achieved in previous studies using etoposide). Additionally, mice treated with 680 μM had a significant survival benefit when compared to control mice, with a 75% cure rate. **(B)** Graphic illustrating different doses of etoposide delivered in this study; 4 μM, 80 μM, 680 μM. **(C)** Mice treated with 680 μM of etoposide using CED also demonstrated lower bioluminescence signal, measured by using bioluminescence imaging to monitor luciferase signaling. *Figures reproduced with permission from Oxford University Press*.

In order to circumvent these challenges, there have been a number of attempts to optimize chemotherapeutic delivery to the CNS. Attempts of using alternative delivery methods like Convection Enhanced Delivery (CED) have yielded promising results. A recent study by our group demonstrated direct intratumoral delivery of high concentrations of etoposide and increased anti-tumor effects against the proneural subtype of GBM (25). In this study, we found that intratumoral delivery of etoposide at a 4uM concentration, which is similar to what is achieved following intravenous delivery, only led to transient decrease in tumor growth with no effect on survival. Yet, a concentration of 80 μM of etoposide delivered intra-tumorally led to a robust survival benefit for transgenic mouse models of proneural gliomas, a subtype of glioma that has been shown to highly express TOP2A and TOP2B (5, 25). Direct intra-tumoral delivery of 680 μM led to cure of most treated mice, and remained well tolerated (Figure 3) (25). It is important to recognize that this study does not establish a causal link between the proneural gene signature with etoposide susceptibility. There is, in fact, causal evidence linking other genes with etoposide susceptibility, and ultimately, there may be better biomarkers to predict etoposide response than the proneural gene signature.

Other attempts to optimize doxorubicin delivery include the use of liposomes (32), nanoparticles (33, 34), focused ultrasound (35), minicells (36), and direct injection (37). In a rat model, MRI-guided focused ultrasound was demonstrated to achieve intratumoral doxorubicin concentrations of 886 ± 327 ng/g tissue which is within the therapeutic range of 819 ± 482 ng/g tumor compared with the control intratumoral concentration of 215 ± 119 ng/g tissue regardless of the dose administered (35).

Combining TOP2 poisons with other agents is another attempt to increase the narrow therapeutic window of these drugs as certain combinations can enhance cytotoxicity as well as increase selectivity (32, 38, 39). Nanoliposomal topotecan in combination with pegylated liposomal doxorubicin administered through CED was found to be associated with a significantly increased median survival, and an additive effect was observed between these two agents in a rodent model (40). Additionally, in certain instances combination therapy may allow for intermittent rather than continuous dosing regiments, allowing for better tolerability while maintaining a similar efficacy. For example, poly (ADP-ribose) polymerase (PARP) inhibition is typically necessary throughout DNA damage and repair processes. However, when combined with TOP2 poisons, which are DNA damaging agents, continuous PARP inhibition may not be necessary as long as a critical inhibitory level is met during only DNA repair (41). This combination of TOP2 poison and PARP inhibitors has been studied in ovarian cancer; a phase 1 dose escalation study with pegylated liposomal doxorubicin in combination with olaparib, a PARP inhibitor, demonstrated that this combination was generally well tolerated, with only 3 out of 44 patients demonstrating dose-limiting toxicities, while 33% of the patients responded to therapy (41).

### Ongoing trials testing TOP2 drugs for GBM

There are currently four clinical trials investigating doxorubicin's role in the treatment of GBM. Some are investigating doxorubicin in the setting of novel delivery mechanisms such as Laser Interstitial Thermal Therapy (LITT) mediated disruption of the BBB (ClinicalTrials.gov-NCT01851733), and nanoparticle delivery targeting cells using bispecific antibodies (ClinicalTrials.gov-NCT02766699). Another is studying the safety and efficacy of aldoxorubicin in subjects with unresectable GBM whose tumors have progressed following prior treatment with surgery, radiation, and temozolomide (ClinicalTrials.gov-NCT02014844). Another trial is investigating the safety and efficacy of prolonged administration of doxorubicin in combination with radiotherapy, temozolomide, and histone deacetylase inhibitor valproic acid in pediatric and adult patients with newly diagnosed GBM and diffuse intrinsic pontine glioma (ClinicalTrials.gov-NCT02758366).

A number of clinical trials studying the etoposide's utility are also ongoing. One trial is investigating combination therapy with sodium thiosulfate in the treatment of gliomas in order to determine if the addition of sodium thiosulfate can protect against chemotherapy related thrombocytopenia (ClinicalTrials.gov-NCT00075387). Another clinical trial is studying the side effects and optimal method of delivering vorinostat with isotretinoin and chemotherapy including IV etoposide phosphate and other agents like carboplatin, cisplatin, cyclophosphamide, thiotepa, vincristine sulfate, and vorinostat against embryonal CNS tumors like medulloblastoma and pineoblastoma. There is a trial investigating the use of LITT to disrupt peritumoral BBB to enhance delivery and efficacy of therapeutic agents including etoposide in the treatment of pediatric brain tumors (ClinicalTrials.gov-NCT02372409). Another clinical trial is investigating overall survival after administration of various chemotherapeutic agents (including etoposide) followed by autologous peripheral stem cell transplantation in the treatment of numerous solid and CNS tumors (ClinicalTrials.gov-NCT01505569).

## Topoisomerase poison susceptibility: biomarkers to personalize therapy

**Figure 4:**
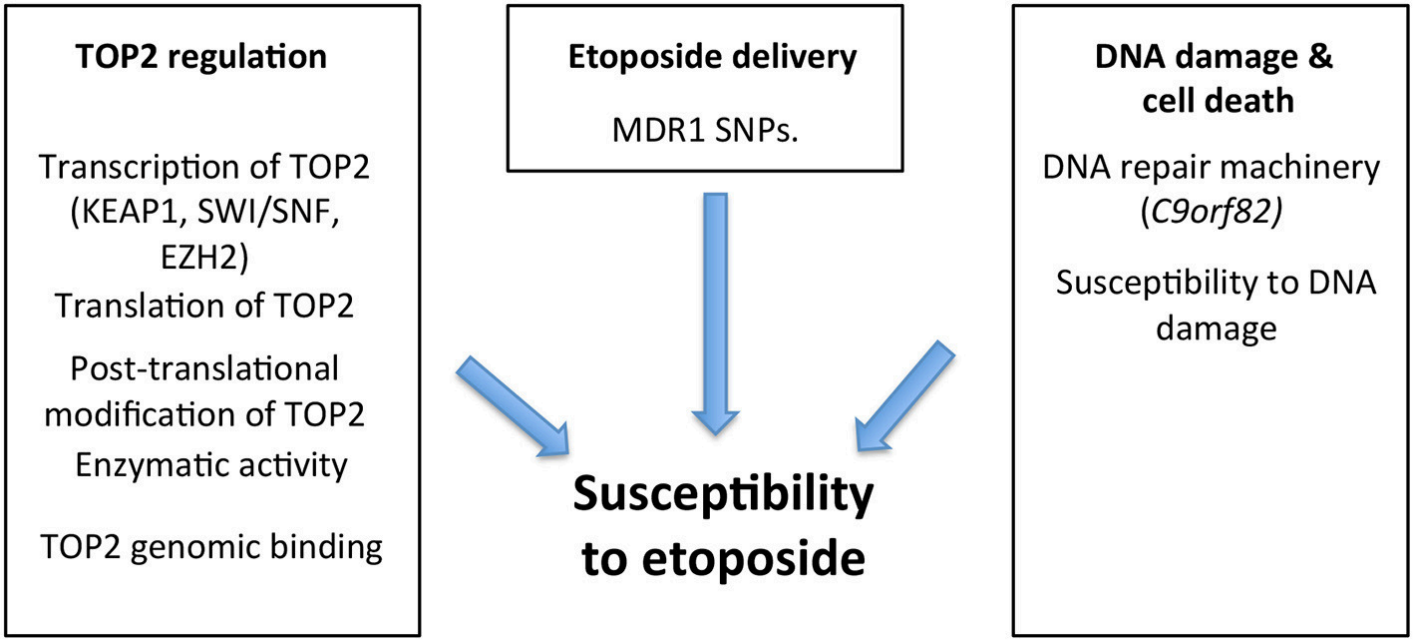
There are several processes that might modulate cancer susceptibility to etoposide, including levels of TOP2 expression (determined by transcription and translation), genomic binding of TOP2 as well as its enzymatic activity and post-translational modifications. Mutations in KEAP1, SWI/SNF complex, and EZH2 influence TOP2 at a transcriptional level, consequently influencing TOP2 resistance/susceptibility. Additionally, etoposide delivery is influenced by resistance proteins like the MDR1 efflux pump, which dictate intracellular concentration and therefore cytotoxicity. The cytotoxic effect of these agents largely stems from their ability to stabilize double-stranded breaks (DSB) in DNA, triggering cellular apoptosis. Therefore, the cell's existing DNA repair machinery, and its overall susceptibility to DNA damage plays a role in determining the success of TOP2 poisons such as etoposide and mutations such as those in *C9orf82* have been found to influence this (43, 44).

For many of the 1 million patients treated annually with a TOP2 poison, therapy is often ineffective and can lead to a number of side effects (16). Many tumors are either initially resistant to these therapies, or become resistant at time of relapse (42). Recognizing underlying mechanisms of resistance/susceptibility (Figure 4) and their tumor molecular signature presents an opportunity to personalize our treatments, and expand our currently narrow therapeutic window by increasing the benefit of these drugs while decreasing unnecessary side effects. The studies that have implicated these underlying mechanisms in susceptibility can be split into two broad categories. The first category, called “experimental” evidence, includes studies in which the gene was experimentally disrupted, leading to cancer cell resistance/susceptibility. The second category, “correlative” evidence, includes genes that are implicated as their natural disruption or variations of expression in some tumors correlates with susceptibility (Table 1).

**Table 1:** This table shows curation of genes identified in **(A)** etoposide /doxorubicin resistance or **(B)** susceptibility, their mechanism of action, means of experimental validation of the genes, correlation to survival, cancers in which they were identified, and the associated reference.

**Gene**	**Mechanism of involvement**	**Expression confers**	**Experimental evidence**	**Correlative evidence**	**Cancer type**	**Which drug**	**Reference**
**ETOPOSIDE RESISTANCE**							
KEAP1	SWI/SNF chromatin remodeler, Top2 expression regulator	Resistance	RNAi	No	Melanoma	Etoposide, Doxorubicin, Daunorubicin, Aclarubicin	(16)
MDR1	Drug transporter/ single nucleotide polymorphism	Resistance	Genotyping, Association studies	Yes-Human	Small cell lung cancer	Etoposide, Cisplatin	(45)
C9OrF82	DNA damage response	Resistance	CRISPR cas9a	No	Melanoma	Etoposide, Doxorubicin, Daunorubicin, Aclarubicin	(16)
SMARCAB1	Chromatin remodeler	Resistance	CRISPR cas9a	No	Melanoma	Etoposide, Doxorubicin, Daunorubicin, Aclarubicin	(16)
SMARCAE1	Chromatin remodeler	Resistance	CRISPR cas9a	No	Melanoma	Etoposide, Doxorubicin, Daunorubicin, Aclarubicin	(16)
ElF4a1	Translation initiation	Resistance	CRISPR cas9a	No	Melanoma	Etoposide, Doxorubicin, Daunorubicin, Aclarubicin	(16)
ABCB1	Multidrug transporter	Resistance	CRISPR cas9a	No	Melanoma	Etoposide, Doxorubicin, Daunorubicin, Aclarubicin	(16)
CHEK2	Cell cycle regulator	Resistance	RNAi	Yes- Mouse model	Lymphoma	Doxorubicin	(42)
TP53	Genome maintenance	Resistance	RNAi	Yes- Mouse model	Lymphoma	Doxorubicin	(42)
TOP2A	DNA unwinding	Resistance	RNAi	Yes- Mouse model	Lymphoma	Doxorubicin	(42)
TOP2B	DNA unwinding	Resistance	RNAi	Yes- Mouse model	Lymphoma	Doxorubicin	(42)
**ETOPOSIDE SUSCEPTIBILITY**							
SEMA5A	Channel protein	Susceptibility	GWAS	Yes- Human	Lymphoblastoid cell line	Etoposide	(46)
SLC7A6	Solute carrier	Susceptibility	GWAS	Yes-Human	Lymphoblastoid cell line	Etoposide	(46)
PRMT7	N methyltransferase enzyme	Susceptibility	GWAS	Yes-Human	Lymphoblastoid cell line	Etoposide	(46)
UVRAG	DNA damage response	Susceptibility	GWAS	Yes-Human	Lymphoblastoid cell line	Etoposide	(46)
ARID1A	SWI/SNF remodeler, inhibition sensitizes to Etoposide	Resistance	RNAi, microarray	Yes-Human	H1975, H2030, HCC4006, A549, HCC2450, PC9, Calu1, H1650, H522, H2126, H157, H1299, HCC15, HCC827, H322, H2009, Sw1573, Calu6, H441, HCC95, H520, H460, Calu3, H2122, H23, H3255	Etoposide	(47)
EZH2	Polycomb repressor complex	Resistance	RNAi, microarray	Yes-Human	H1975, H2030, HCC4006, A549, HCC2450, PC9, Calu1, H1650, H522, H2126, H157, H1299, HCC15, HCC827, H322, H2009, Sw1573, Calu6, H441, HCC95, H520, H460, Calu3, H2122, H23, H3256	Etoposide	(47)

### Levels of expression of TOP2 protein

A number of studies have thus far shown an association between levels of topoisomerase expression and cellular susceptibility to TOP2 poisons (48, 49). TOP2A suppression is thought to result in resistance by decreasing the amount of enzyme-DNA complex, in turn decreasing the amount of DNA damage (42). Our group found a correlation between the transcript levels of TOP2B and etoposide susceptibility across 139 human cancer cell lines (25). We demonstrated that TOP2A levels were significantly elevated in platelet-derived growth factor (PDGF)^+^ and phosphatase tensin homolog (PTEN)^−/−^ mouse proneural tumors which were susceptible to intratumorally delivered etoposide by CED (correlative evidence) (Figure 3) (25). Several additional studies have demonstrated that TOP2 poison susceptibility is related to levels of TOP2 expression. One particular study decreased levels of TOP2A expression using gene suppressor elements (GSE's) and demonstrated a resistance to etoposide in different mammalian cell lines (experimental evidence) (50). Another study explored the genetic basis for response heterogeneity using a pool-based RNAi screening approach, identifying TOP2A expression levels as major determinants of doxorubicin response in a mouse model of lymphoma. In addition, by decreasing TOP2A expression *in vivo* using retrovirally encoded shRNAs (experimental evidence), this study was able to demonstrate a relationship between TOP2A levels and doxorubicin susceptibility (42). This same study noted that tumors which relapsed after doxorubicin therapy displayed highly reduced TOP2A levels (42).

Merely suppressing the expression of TOP2A and demonstrating resistance does not prove that TOP2A expression levels can be used as a biomarker to personalize therapy. Rather, this only succeeds in demonstrating etoposide and doxorubicin's mechanisms of action. The more important question is whether or not naturally occurring variations in TOP2A expression correlate with etoposide/doxorubicin susceptibility, which does not (25). This implies that TOP2A expression is necessary but perhaps not sufficient for efficacious tumor cell killing by these agents. It is possible that there are other molecular factors limiting the effectiveness of these agents, and that even a small amount of TOP2A expression is sufficient to elicit DNA damage if other conditions are met. Importantly, many studies have demonstrated that increased TOP2A expression is correlated with improved prognosis in GBM, measured as survival at 2 years (51), 5-year progression free survival and overall survival (52, 53) (correlative evidence).

### Other genes associated with susceptibility to TOP2 poisons

#### KEAP1

Mutations in the KEAP1 (Kelch-like ECH-associated Protein-1) gene are common abnormalities in non-small cell lung cancer (NSCLC), gallbladder, liver (54), ovarian (55), endometrial (56), and lung papillary cancers (57). Keap1 is an E3 ubiquitin ligase involved in degrading Nrf2, which regulates transcription of genes that mediate the response to oxidative stress (58). Studies have suggested that decreased Keap1 expression and increased Nrf2 expression enhances tumor cell growth. A systematic analysis of the KEAP1 genomic locus in NSCLC cell lines demonstrated bi-allelic inactivation in KEAP1 was associated with constitutive activation of Nrf2-mediated gene expression (59). Another study demonstrated similar findings in biliary tract and gallbladder cancers. Amplifying alterations in KEAP1 are found in approximately 0.8% of GBM tumors (43, 44). Mutations in the Keap1-Nrf2 pathway have been implicated in TOP2 poison resistance (60). For instance, Wang, et. al demonstrated that stable overexpression of Nrf2 resulted in increased resistance to agents such as etoposide, cisplatin, and doxorubicin. Conversely, they demonstrated down-regulation of the Nrf2 response was associated with increased susceptibility to the above therapies (experimental evidence) (61). Also, a recent study used a genome wide knock down approach and demonstrated that KEAP1 mutations also confer resistance to TOP2 poisons by decreasing TOP2A expression levels (experimental evidence) (16). Therefore, assessing the mutational status of KEAP1 prior to initiating therapy may provide a more informed and personalized approach to treatment.

#### ABCB1

Drug efflux is a well-researched mechanism of resistance to chemotherapy, where increased activity of a molecular pump decreases the intracellular accumulation of a therapeutic agent (62). In particular, three such transporters—multidrug resistance protein 1 (MDR1), multidrug resistance associated protein 1 (MRP1), and breast cancer resistance protein (BCRP) have been implicated in cancer resistance to a number of chemotherapeutic agents (62). MDR1 encodes P-glycoprotein, a protein that functions as an active efflux pump, removing lipophilic compounds from the cell, thus decreasing the intracellular accumulation of chemotherapeutics. Many studies have demonstrated a negative correlation between P-glycoprotein and chemotherapeutic efficacy (correlative evidence) (63–65). This protein is especially important for the treatment of neurological malignancies as it removes drugs at the BBB, preventing access of agents to the CNS (16). Animal studies have demonstrated that specifically inhibiting P-glycoprotein can increase the level of drug in the brain while also improving the drug's efficacy against implanted human tumors (66, 67). However, later clinical attempts to incorporate P-glycoprotein inhibitors into chemotherapy protocols have shown contradictory and disappointing results (68–70). This discrepancy may be explained by suboptimal dosing of both the inhibitor and chemotherapeutic, with possible contribution by other pumps on the luminal side of brain capillary endothelial cells (71). The presence of P-glycoprotein may be an important molecular biomarker whose presence should be assessed before starting therapy with a TOP2 poison, however, studies are yet to determine a conclusive clinical benefit to targeting these resistance mechanisms.

#### C9Orf82

The activity of C9orf82 been recently implicated in resistance to TOP2 poisons. There is currently no consensus as to its precise function in the cell, with some data suggesting it negatively regulates apoptosis mediated by caspases (72), and other data suggesting it is involved with the repair of DNA after DSB induced by TOP2 proteins (experimental evidence) (16). Alterations in C9orf82, predominantly deletions, are found in approximately between 6 and11% of GBM tumors (43, 44). A mutation in the C9orf82 gene was shown to confer resistance to TOP2 poisons (experimental evidence) (16). The role of C9orf82 needs to be examined further before its clinical use as a reliable biomarker.

#### SWI/SNF complex

The SWI/SNF complex is involved in regulating gene expression via chromatin remodeling. This complex controls DNA accessibility to transcription factors by altering histone-DNA interactions in an ATP-dependent manner (73, 74). This complex is composed of a number of subunits, including SNF5/SMARCB1, SMARCE1, BRG1, PBRM1/BAF180 (75). The SWI/SNF complex has been implicated in malignant rhabdoid tumors (76), and specific subunits of this complex (SNF5/SMARCB1, SMARCE1, BRG1, PBRM1/BAF180) have been identified in approximately 20% of human cancers including renal carcinoma (77), and NSCLC (78). Alterations in individual components of the SWI/SNF complex are also seen in GBM, with mutation, fusion, deletion, or amplification occurring in between 0.25 and 3.5% of tumors depending on the subunit and the underlying gene (43, 44). This complex can influence susceptibility to TOP2 inhibitor therapy as units like SMARCB1 are involved in loading TOP2A onto DNA, and thus can determine how many DNA breaks a cancer cell will have when exposed to a TOP2 poison (16). This is consistent with *in vitro* findings (experimental evidence) (16) and clinical observations, as malignant rhabdoid tumors are unresponsive to doxorubicin (79).

#### EZH2

EZH2 (Enhancer of Zeste Homolog-2) is another chromatin remodeler implicated in cancer resistance to chemotherapy (80). This molecule is a subunit of a larger complex called the Polycomb Repressive Complex 2 which is involved in the tri-methylation of histone 3 at lysine 27 to negatively regulate transcription (81). Evidence implicating EZH2 in oncogenesis exists in a variety of cancer types including prostate, breast cancer, melanoma, and bladder cancer (82, 83). EZH2 has been implicated in the sensitivity/resistance to TOP2 poisons; NSCLC cell lines have demonstrated enhanced sensitivity to EZH2 inhibition with etoposide therapy both *in vitro* and *in vivo* (47).

## Conclusion

As the prognosis of patients diagnosed with GBM remains poor, further efforts should be dedicated to improving the efficiency of our medical therapies. The genetic heterogeneity of GBM cell lines presents a unique opportunity to investigate possible biomarkers to personalize therapy. A precision medicine approach to chemotherapy for brain tumors can potentially enhance efficacy of these treatments, and avoid unnecessary exposure to toxic agents that are not helpful for some cases. The use of TOP2 poisons is an apt illustration of this opportunity, with a growing body of research identifying biomarkers and unique tumoral characteristics that influence susceptibility. Additionally, the use of novel delivery techniques may allow us to achieve therapeutic intratumoral concentrations of TOP2 poisons without having to administer potentially toxic systemic doses, which up to this point has limited their effectiveness. Together, these offer a glimpse into precision therapy and personalized medicine, allowing us to enhance the efficacy of existing therapies in efforts to make progress in a disease that has proved extremely difficult to treat.

## Author contributions

AM contributed to the writing and reviewing of the manuscript, reviewing the literature, editing the manuscript, and submitting the manuscript. CA contributed to the editing and writing of the manuscript, contributing scientific expertise, conducting original analysis based on existing data. AS contributed to the overall vision behind the manuscript, writing the manuscript, editing the manuscript, supervising all other aspects of its creation, and contributing scientific expertise.

### Conflict of interest statement

The authors declare that the research was conducted in the absence of any commercial or financial relationships that could be construed as a potential conflict of interest.
